# Serum Levels of Soluble CD40 Ligand and Neopterin in HIV Coinfected Asymptomatic and Symptomatic Visceral Leishmaniasis Patients

**DOI:** 10.3389/fcimb.2018.00428

**Published:** 2018-12-11

**Authors:** Wim Adriaensen, Saïd Abdellati, Saskia van Henten, Yonas Gedamu, Ermias Diro, Florian Vogt, Bewketu Mengesha, Emebet Adem, Luc Kestens, Johan van Griensven

**Affiliations:** ^1^Unit of NTDs, Department of Clinical Sciences, Institute of Tropical Medicine, Antwerp, Belgium; ^2^Department of Internal Medicine, Leishmaniasis Research and Treatment Centre, University of Gondar, Gondar, Ethiopia; ^3^Unit of Immunology, Department of Biomedical Sciences, Institute of Tropical Medicine, Antwerp, Belgium

**Keywords:** visceral leishmaniasis, kala-azar, HIV, sCD40L, neopterin, asymptomatic

## Abstract

Human Immunodeficiency Virus (HIV) co-infection drastically increases the risk of developing overt visceral leishmaniasis (VL). The asymptomatic *Leishmania* infection window constitutes an opportunity to identify those HIV patients at highest risk by defining early markers associated with disease susceptibility or resistance. As intracellular parasite killing is essential, we investigated whether serum markers of macrophage activation were notably affected in HIV patients with an asymptomatic *Leishmania* infection or overt visceral leishmaniasis disease. Serum levels of soluble CD40 ligand and neopterin were assessed in 24 active VL-HIV patients, 35 HIV patients with asymptomatic *Leishmania* infection and 35 HIV endemic controls. All patients were recruited in *L. donovani* endemic regions of North-West Ethiopia. The serum levels of sCD40L and neopterin significantly decreased and increased in HIV patients with active VL compared to HIV patients with asymptomatic *Leishmania* infection, respectively. No statistically significant differences could be detected in neopterin and sCD40L levels between *Leishmania* asymptomatically infected HIV patients and endemic HIV control patients. However, an inverse trend, between *Leishmania* antibody positivity or VL development and neopterin levels could be seen. The CD4+ T-cell count was inversely correlated with serum neopterin levels, but not with sCD40L levels. Our results in HIV coinfected patients, correspond with the postulated protective role of sCD40L in VL and underline the importance of the CD40-CD40L pathway in resistance against the parasite. Neopterin levels suggest an increased macrophage activation upon infection and could have a value in clinical algorithms to, although non-specifically, improve prediction of VL development in HIV patients with asymptomatic *Leishmania* infection.

## Introduction

Human Immunodeficiency Virus-1 (HIV-1) has been identified as one of the emerging challenges for Visceral Leishmaniasis (VL) control, an important yet neglected vector-borne disseminated infection caused by the protozoan *Leishmania donovani* spp. complex (van Griensven et al., 2014b). The anthroponotic form of VL is caused by *Leishmania donovani* and is prevalent in the Indian subcontinent (300,000 cases/year) and East Africa (30,000 cases/year), mainly Sudan and Ethiopia (van Griensven and Diro, 2012). Untreated, overt disease is universally lethal. HIV is one of the strongest risk factors to develop VL. In contrast with the zoonotic *Leishmania infantum* endemic regions in Europe, where introduction of anti-retroviral therapy (ART) resulted in a significant reduction in the incidence of VL-HIV (Desjeux and Alvar, 2003), scaling-up of ART did so far not yield similar effects in East-Africa. In Ethiopia, close to 30% of patients with VL are co-infected with HIV, and an increasing number and proportion of VL cases are now seen in individuals on ART, including primary VL episodes (Diro et al., 2014). In addition, once the infection has evolved to active VL in HIV patients (typically within 6–9 months after infection) it is characterized by low cure rates, higher drug toxicity, frequent VL relapse and high case-fatality rates (van Griensven et al., 2010). The asymptomatic *Leishmania* infection window constitutes an opportunity to define early markers associated with disease control or progression (van Griensven et al., 2014a). To date, our knowledge on early immunopathology of VL is limited, and very scarce in HIV coinfected patients (Okwor and Uzonna, 2013). As both infections are clearly associated with immune deficiency, simple serum markers of a deteriorating immune response may allow an early detection of those at high risk for progression to VL. Chronic immune activation is a typical characteristic of HIV disease progression and several biomarkers also proved informative for coinfection progression (Sokoya et al., 2017). In a similar manner, VL showed to be an independent source of chronic immune activation in VL-HIV patients (Casado et al., 2015). Therefore, we investigated whether serum markers of immune activation, in particular macrophage activation, were notably affected in HIV patients with an asymptomatic *Leishmania* infection and overt VL disease.

One marker of interest is CD40 ligand (CD40L or CD154). This membrane glycoprotein is primarily expressed on activated CD4+ T-cells, platelets and a small proportion on CD8+ T-cells (Kornbluth, 2000). It binds and activates CD40 on antigen presenting cells, thereby enhancing the survival of the APC and promotes secretion of pro-inflammatory cytokines and synthesis of nitric oxide (NO) (Subauste, 2009). A T-helper 1 (Th1) cell-mediated immune response with high interferon(IFN)-y production activating macrophages to produce NO is reported to be protective in *Leishmania*-infected murine models (Rodrigues et al., 2016). Vice versa, a Th2-skewed response with high levels of IL-10 was shown to be detrimental. This dichotomy is not so clear in human VL patients, let alone in HIV coinfected patients (McMahon-Pratt and Alexander, 2004). Irrespective, the production of both IL-10 and IFN-y is dependent on this costimulatory pathway. Several studies in mice and human lymphocytes underlined the central role of the CD40-CD40L pathway in the generation of effective T-cell responses and protection against *Leishmania* and other parasitic infections (Subauste, 2009).

sCD40L, the soluble derivate of CD40L, is a functional trimer which retains its biological function after cleavage of the T-cell membrane, allowing it to interact with and activate cells expressing CD40, such as macrophages. This soluble form was associated with clinical resolution of VL (de Oliveira et al., 2013). On top of a gradual increase in serum sCD40L levels during treatment, levels were also negatively correlated with spleen size and parasite load. The same authors recently showed that sCD40L from sera of exposed subjects could indeed increase production of inflammatory cytokines and improve control of the parasite in human *L. infantum* infected macrophages (de Oliveira et al., 2015). The variation in sCD40L levels and its prognostic value in asymptomatic *Leishmania* infection or a concurrent HIV coinfection is unknown.

Increased serum levels of neopterin are associated with immune activation and showed to be one of the better soluble predictors of adverse outcomes in HIV patients (disease progression, ART activity or inflammation-associated comorbidities), at least comparable to that of the number of CD4+ T-cells (Nyamweya et al., 2012; Eisenhut, 2013; Bipath et al., 2015). Neopterin is a purine nucleotide derivate from guanosine triphosphate (GTP) and produced by human and primate IFN-y-activated macrophages (Hamerlinck et al., 2000). Hence, neopterin levels are increased in pathologies associated with a Th1 dominated immune response and usually correlate well with the disease stage. The fact that neopterin is produced by the common target cell of *Leishmania* and HIV (cf. macrophage), we investigated whether neopterin levels can be used as a marker of T-cell activation and produced oxidative stress inducing intracellular *Leishmania* parasite killing in HIV patients. Because previous studies have reported increased neopterin levels in the early phases of viral infections (e.g., EBV, CMV, and parvovirus B19), we investigated the asymptomatic *Leishmania* infection phase in particular (Reibnegger et al., 1988; Murr et al., 2002).

This study is the first to assess the association of serum sCD40L and neopterin concentrations with asymptomatic and symptomatic *Leishmania* infection status in HIV patients living in VL-endemic regions.

## Methods

### Study Design and Population

All patients were recruited in *L. donovani* endemic regions of North-West Ethiopia (Abdurafi, Metema and Gondar). Active VL-HIV patients were selected from a pentamidine secondary prophylaxis clinical trial for VL relapse in HIV coinfected patients (NCT01360762) in which 24 patients had available serum samples from their baseline visit (before initial treatment). Baseline samples from 35 asymptomatic *Leishmania* antibody positive HIV patients and 35 endemic HIV controls with no antibodies against *Leishmania* were selected from an observational cohort study on asymptomatic *Leishmania* infection in HIV patients (NCT02839603). All 34 of 35 asymptomatic *Leishmania* antibody positive HIV patients remained disease free for a median of 12 months (IQR: 9–12) and one patient developed VL 9 months later. In contrast to CD4+ T-cell counts < 200 cells/mL in the majority of active VL-HIV cases, we expected higher heterogeneity in HIV history and CD4+ T-cell counts in non-diseased *Leishmania* antibody positive and negative HIV patients that could affect sCD40L and neopterin serum concentrations. For this reason, non-diseased individuals with and without antibodies against *Leishmania* were individually matched on sex, months on ART, ART regimen, and CD4+ T-cell count. Antibody positivity was tested with rK39-Rapid diagnostic test (RDT) (Kalazar Detect Rapid Test, InBios International Inc., Seattle).

### Serum Markers of Macrophage Activation

Concentrations of human neopterin were measured by enzyme immunoassay (ELISA, IBL international, Germany), with an upper limit of 29,400 pg/mL (no left-over sample for further dilution). Likewise, concentrations of human sCD40L were measured in serum samples by enzyme immunoassay (ELISA, IBL international, Germany).

### Covariates

An antibody-detecting direct agglutination test (DAT, Institute of Tropical Medicine, Antwerp) was performed on all serum samples and a titer ≥1:200 was considered positive in case of asymptomatic infection and ≥1:6,400 in case of active VL-HIV patients. Three VL-HIV patients had missing DAT values. Urine samples were used to perform the KAtex urine antigen test (Kalon Biological Ltd, Guildford, UK). Four VL-HIV patients had missing KAtex values. Microscopy for malaria and parasitic infections was performed in whole blood and stool samples, respectively.

### Statistical Analyses

Continuous data are presented as medians and interquartile ranges (IQR). Categorical data are presented as numbers and frequencies. Comparisons between asymptomatic *Leishmania* antibody positive cases and active VL-HIV cases were performed using the chi-square test and Mann–Whitney *U-*test for continuous data. Comparisons between the matched HIV patients with and without *Leishmania* antibody positivity were performed using robust conditional logistic regression and McNewar Chi2 test. *p* < 0.05 was considered to be statistically significant. Dot plots are shown with median and IQR. Spike curves showed the individual change from case to control in each matched pair. Correlations between CD4+ T-cell counts and our markers of interest were plotted and the corresponding Pearson correlation coefficients were calculated. The statistical analyses were performed using STATA 14 (StataCorp, College Station, TX, United States) and GraphPad Prism 7 (GraphPad Software, San Diego, CA, United States).

## Results

The matched case-control study consisted of 70 HIV patients living in a VL endemic area in North-West Ethiopia, 50% with confirmed positive antibody test against rK39 antigen. All 34 of 35 asymptomatic *Leishmania* antibody positive HIV patients remained disease free for a median of 12 months (IQR: 9–12) and one patient developed VL 9 months later. Of all cases and controls, 78.6% lived in the endemic area for more than 10 years and showed potential risk factors for *Leishmania* infection, with 66 (94.3%) having animals in or around the house, 49 (70%) were sleeping outside and most patients were male (85.7%) daily laborers of farmers working on the fields (78.3%) (Table 1). With respect to their HIV infection, the majority were on ART (91.4%) for more than 2 years (60%) with fairly good CD4+ T-cell counts (Table 1). In general, patients were rather malnourished with 40% having a body mass index below 18.5 kg/m^2^; 14 (20.6%) had intestinal parasites in their stool.

**Table 1:** Patient characteristics.

	**HIV patients (*****n*** **=** **70, matched design)**		*p*-value*	**VL-HIV patients (*n* = 24)**	*p*-value**
	**rK39– (*n* = 35)**	**rK39+ (*n* = 35)**		
**DEMOGRAPHICS, n(%)**					
Age category, years			0.796		0.036
18-27	7 (20)	3 (8.6)		8 (33.3)
28-37	12 (34.3)	18 (51.4)		13 (54.2)
38-47	9 (25.7)	11 (31.4)		2 (8.3)
>47	7 (20)	3 (8.6)		1 (4.2)
Male***	30 (85.7)	30 (85.7)	1.000	23 (95.8)	0.206
Occupation			0.673	Not collected
Farmer	19 (54.3)	19 (54.3)		
Daily laborer	6 (17.1)	10 (28.6)		
Merchant	6 (17.1)	0 (0)		
Housewife	2 (5.7)	3 (8.6)		
Other	1 (2.9)	3 (8.6)		
*Missing*	1 (2.9)	0 (0)		
**CLINICAL DATA, n(%)**					
Not on ART***	3 (8.6)	3 (8.6)	1.000	12 (50)	< 0.001
Time on ART, months***			1.000		0.016
< 24	11 (34.4)	11 (34.4)		9 (75)
≥24	21 (65.6)	21 (65.6)		3 (25)
CD4 category, cells/mL***			1.000		0.001
< 350	16 (45.7)	16 (45.7)		22 (91.7)
350 to < 500	12 (34.3)	12 (34.3)		2 (8.3)
≥500	7 (20)	7 (20)		0 (0)
Body mass index, kg/m^2^			0.675		0.058
< 18.5	14 (40)	14 (40)		17 (70.8)
18.5- < 25	18 (51.4)	20 (57.1)		7 (29.2)
≥25	3 (8.6)	1 (2.9)		0 (0)
Intestinal parasites	5 (15.2)	9 (25.7)	0.227	Not collected
Malaria infection	2 (5.9)	3 (8.6)	1.000	Not collected
**MARKERS OF EXPOSURE, n(%)**					
Previous VL	0 (0)	5 (14.3)	0.025	13 (54.2)	0.001
DAT positive	0 (0)	15 (42.9)	< 0.001	20 (95.2)	< 0.001
KAtex positive	0 (0)	1 (2.9)	0.317	16 (80)	< 0.001
Animals present in or around the patient house	33 (94.3)	33 (94.3)	1.000	Not collected
Sleeping outside at home	23 (65.7)	26 (74.3)	0.448	Not collected
Time patient living in endemic area ≥10 years	30 (85.7)	25 (71.4)	0.277	Not collected

* *p-value comparing the non-infected cases against the asymptomatic cases by robust conditional logistical regression (continuous variables) or McNemar Chi2 test (categorical variables)*.

** *p-value comparing the active cases (24 VL-HIV patients) against the asymptomatic cases (35 rK39+ patients) by chi2-test*.

*** *Matching criteria*.

Of the 35 *Leishmania* antibody positive asymptomatic cases, five (14.3%) had a previous VL episode (Table 1). Fifteen (42.9%) patients also tested antibody positive on DAT in serum and only one tested rK39 antigen positive on a latex agglutination test (KAtex) in the urine. Besides the infection markers, asymptomatic HIV patients with *Leishmania* antibody positivity were not statistically significantly different from HIV patients without *Leishmania* antibody positivity (Table 1).

**Figure 1:**
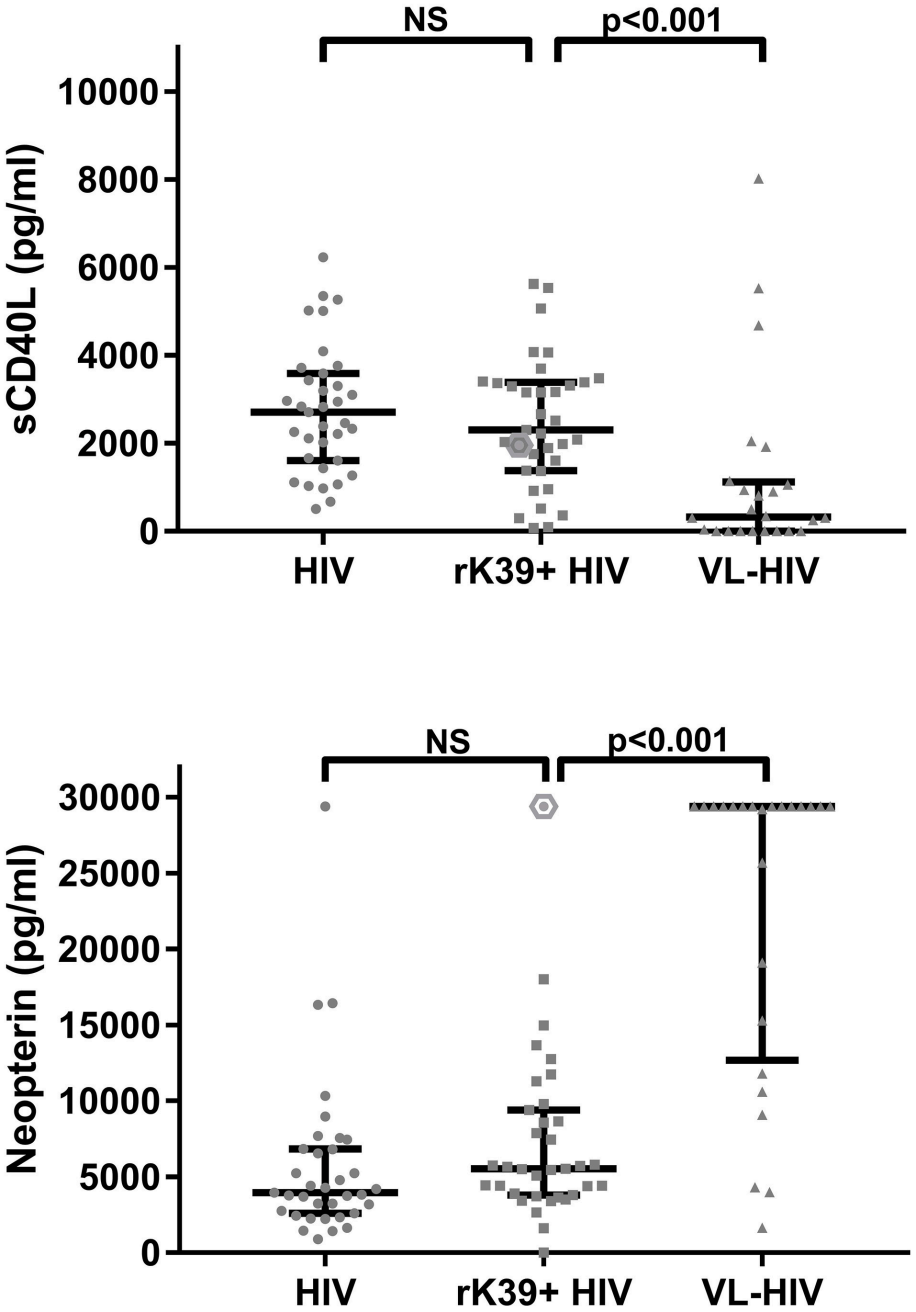
Dot plot of serum sCD40L **(top)** and neopterin **(bottom)** concentrations per *Leishmania* infection status group in HIV patients. Hexagon indicates single HIV patient that developed VL 9 months later.

Twenty-four HIV patients with overt VL disease from the same region were included in the study. The serum levels of sCD40L and neopterin significantly decreased and increased in active VL-HIV cases [325 pg/mL (IQR: 0–1097.5), 29,400 pg/mL (IQR: 13,550–29,400)] compared to asymptomatic *Leishmania* antibody positive HIV patients [2,490 pg/mL (IQR: 1,605–3,405), 4,610 pg/mL (IQR: 3,430–7,700)], respectively (Figure 1). The single asymptomatic *Leishmania* infected patient that developed VL 9 months later had sCD40L and neopterin concentrations of 1,955 and 29,400 pg/mL, respectively (hexagon in Figure 1). The VL-HIV patients consisted almost exclusively of male patients (95.8%), who were significantly younger (*p* = 0.036), had a lower CD4+ T-cell count (*p* = 0.001), with only 50% on ART (*p* < 0.001) with a shorter time on ART (*p* = 0.016), compared to the asymptomatic *Leishmania* antibody positive HIV patients (Table 1). No difference in serum immune activation markers could be detected in pre-ART and ART patients among active VL-HIV patients (Table 2). In contrast to sCD40L levels, where no association could be detected, neopterin levels were inversely associated with the CD4+ T-cell count (Figure 3).

**Table 2:** Median and interquartile ranges for sCD40L and neopterin levels with regard to ART status in active VL-HIV patients.

	**VL-HIV patients (*****n*** **=** **24)**		*p*-value*
	**Pre-ART (*n* = 12)**	**ART (*n* = 12)**
sCD40L, pg/mL	402.5 (22.5–1,530)	325 (0–977.5)	0.702
Neopterin, pg/mL	29,400 (22,350–29,400)	27,450 (9,840–29,400)	0.147

* *p-value Mann Whitney U–test*.

**Figure 2:**
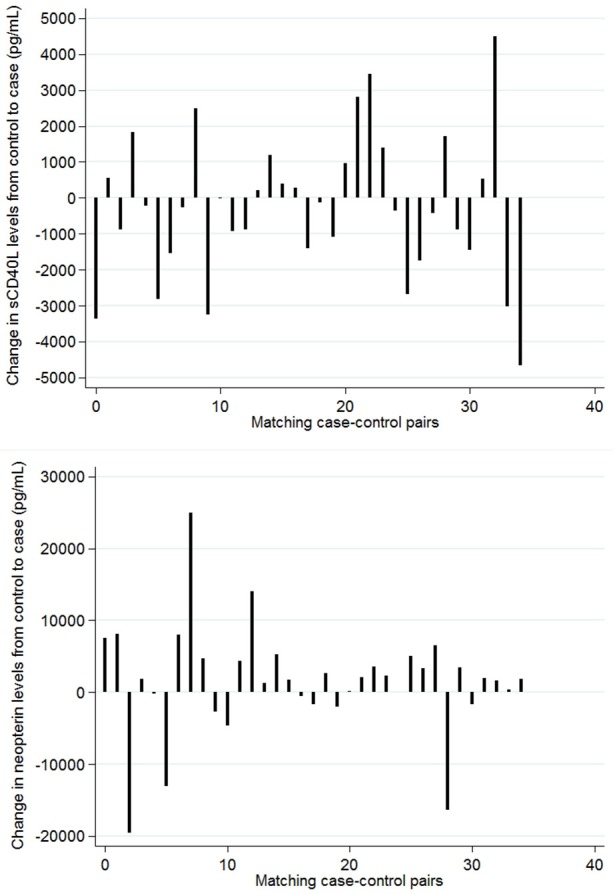
Change in serum sCD40L **(top)** and neopterin **(bottom)** concentrations per matched control-case pair. Controls: rK39– HIV patients Cases: rK39+ HIV patients. Patients were matched on sex, months on ART, ART regime, and CD4+ T-cell count.

To account for the higher heterogeneity in HIV history and CD4+ T-cell counts in non-diseased HIV patients with and without *Leishmania* antibodies, matched analyses were performed to be able to detect small differences due to the parasitic infection in the levels of sCD40L and neopterin independent from the concurrent HIV/ART stage. No significant differences could be detected in sCD40L and neopterin levels between *Leishmania* asymptomatically infected HIV patients and endemic HIV control patients (Figure 1). Because matched analyses were performed, group median values shown in Figure 1 for non-diseased HIV patients with and without *Leishmania* antibodies are less informative. Alternatively, spike curves were plotted to show the changes in levels among case-control pairs (Figure 2). The median change from control to case in serum sCD40L and neopterin levels was −255 pg/mL (IQR: −1,445, 965) and 1,860 pg/mL (IQR: −550, 4,700), respectively. An inverse trend, although not statistically significant, between *Leishmania* antibody positivity and neopterin levels could be observed (Figures 1, 2).

## Discussion

Because the activation of macrophages for intracellular parasite killing is essential with regard to VL progression or resistance, we investigated the association of serum macrophage activation markers with the status of *Leishmania* infection in HIV coinfected patients.

Comparable levels of sCD40L were found in the serum of asymptomatic *Leishmania* antibody positive HIV patients and endemic healthy controls. In the latter, levels were slightly higher compared to previously published endemic healthy controls in Ethiopia (Gadisa et al., 2017). In particular, serum levels of sCD40L were significantly decreased in diseased VL-HIV patients. This is in clear contrast with previous studies that detected high levels of sCD40L in chronic hepatitis C HIV coinfected patients (Lapinski et al., 2014) and suggested high shedding of sCD40L due to T-cell turn-over as a marker of immune activation and disease, associated with T-cell exhaustion and poor prognosis of HIV infection (Kornbluth, 2000; Miller et al., 2015). Higher levels of sCD40L have also been reported in untreated HIV patients than in ART-treated HIV patients (Olmo et al., 2012), but this could not be confirmed in our VL-HIV group (Table 2). Previously published data in VL patients also showed very low sCD40L levels (de Oliveira et al., 2013; Gadisa et al., 2017), supporting the hypothesis of a specific parasite-driven inhibition of the CD40 costimulatory pathway. *L. major* amastigotes were shown to modulate the CD40-CD40L pathway downstream by inducing ERK1/2 and IL-10 production, which inhibits the p38MAPK/IL12 pathway resulting in persistence of infection (Subauste, 2009; de Oliveira et al., 2015). A continuous loop could be created as it has also been reported that IL-10 is among those mediators that reduces sCD40L expression (Daoussis et al., 2004).

**Figure 3:**
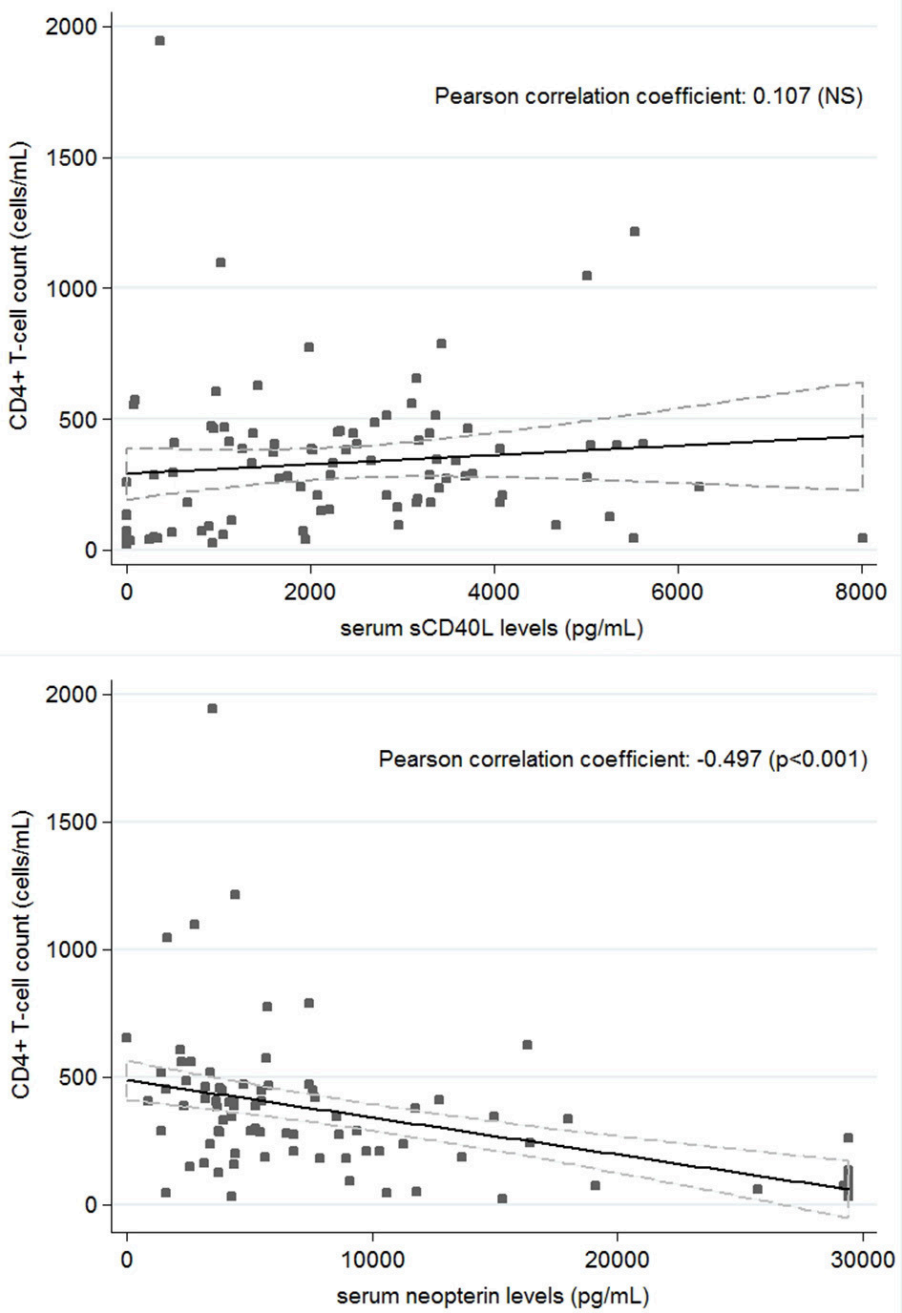
Correlation between CD4+ T-cell count and serum sCD40L **(top)** and Neopterin **(bottom)** concentrations in all patients (*n* = 94).

We believe sCD40L could induce a strong CD4+ T-cell independent activation of macrophages, especially in CD4+CD40L+ T-cell deprived HIV conditions, resulting in IFN-y and NO production followed by parasite clearance. In line with the proposed CD4+ T-cell independent activation, no correlation was found between sCD40L levels and the CD4+ T-cell count in our study population (Figure 3) nor in previous studies among HIV patients (Kalayjian et al., 2010; Lapinski et al., 2014). Moreover, de Oliveira and coworkers recently showed that sCD40L from sera of exposed subjects could indeed increase production of inflammatory cytokines and improve control of the parasite in human *L. infantum* infected macrophages (de Oliveira et al., 2015). In addition, high levels of sCD40L in non-diseased non-HIV individuals living in high risk endemic settings in Brazil (however with unknown infection status), compared to very low levels in non-endemic controls, suggests a protective role of sCD40L in *Leishmania* infection and disease (de Oliveira et al., 2013). We obtained similar results in HIV coinfected controls.

In experimental models of leishmaniasis, strong CD40-CD40L signaling induced IL-12 production by macrophages whereas weak signaling induced IL-10 production (Mathur et al., 2006). The CD40-CD40L interaction thus seems to steer resistance or susceptibility to infection and indicates a potential relevance of sCD40L supplementing in control of the early infection. Although all monomeric, dimeric, and trimeric forms of soluble CD40L can bind to CD40, the soluble trimeric form of CD40L has the most potent biological activity through oligomerization of cell surface CD40 (Manzoor, 2015). For this reason, recombinant trimeric sCD40L could be considered as a potential adjuvant for a therapeutic or prophylactic vaccine approach in HIV patients. The latter is supported by the ability of recombinant sCD40L to potentiate vaccine-induced immunity against *L.major* infection (Gurunathan et al., 1998; Chen et al., 2001). Previous studies in knock out mice and *in vitro* models using recombinant trimeric (s)CD40L or anti-CD40 mAb also showed the important role of the CD40 costimulatory pathway in protection against *L.major* and *L.donovani* infection (Campbell et al., 1996; Soong et al., 1996; Murray et al., 2003; Murray, 2005). Although a larger effect could be anticipated in an HIV population with decreased CD4+CD40L+ T-cells numbers, we cannot extrapolate these findings to HIV individuals with a suppressed immunity as the mechanisms behind the improved parasite killing remain unknown.

In addition, neopterin levels were studied as a marker of the total effect of immunological interactions on the populations of macrophages (Sucher et al., 2010). Neopterin levels are produced upon macrophage activation mainly by IFN-y and to a lesser extent by IFN-α and β, reflecting an activated cellular immunity. Besides an observed increase in active VL-HIV patients due to an activated Th1-mediated immune response and the herewith connected development of oxidative stress against intracellular *Leishmania* parasites, neopterin levels have also been constitutively reported to be elevated in active HIV patients having an increased number of activated CD4+ T-cells which are highly susceptible for HIV infection. Hence, in contrast to sCD40L, neopterin levels seem to reflect inflammation due to HIV and were inversely associated with the CD4+ T-cell count (Figure 3; Chadha et al., 2013). Linked to the CD4+ T-cell count recovery, a decrease in neopterin levels is reported after successful ART although these do not normalize completely in HIV infected individuals (Amirayan-Chevillard et al., 2000; Chadha et al., 2013). As normal serum values of neopterin range until 2,200 pg/ml in healthy people, a median of 3,960 pg/ml fits with a slightly elevated level of neopterin in our population of stable HIV patients on ART. Active VL-HIV patients with a median of 29,400 pg/ml showed comparable levels to newly diagnosed HIV patients (Amirayan-Chevillard et al., 2000) and previous VL patients (Hamerlinck et al., 2000).

Accounting for the CD4+ T-cell count association, no statistically significant difference could be found between non-diseased HIV patients with *Leishmania* antibodies and those without *Leishmania* antibodies in our CD4-matched case-control study, with a small trend toward higher levels in *Leishmania* antibody positive HIV patients (Figures 1, 2). This suggests the lack of a *Leishmania*-specific effect on serum levels of neopterin. Although an increasing number and proportion of VL cases are now seen in individuals on ART, HIV patients with and without *Leishmania* antibodies studied here were rather stable ART patients with high CD4+ T-cell counts, as we selected long-term residents of the study area in stable follow-up at the ART clinic. Respectively, only one male patient presented with VL 9 months after his baseline sampling (see hexagon in Figure 1). Interestingly, this patient had the highest level of neopterin at baseline and a rather average concentration of sCD40L. This single case observation corresponds with the postulated value of neopterin levels to, although non-specifically, predict opportunistic infections in HIV patients but have less value as a specific marker of early *Leishmania* immunopathology in HIV patients. Neopterin production was for instance also reported to be increased in adults with TB-HIV coinfection (Skogmar et al., 2015). Although therefore less sensitive to screen for VL progression in particular, neopterin levels could be a valuable marker in a clinical predictive algorithm for resource-constrained settings. Compared to other cytokines, neopterin also has a higher stability in body fluids allowing easy sample handling. In addition, a rapid test is being constructed and urinary neopterin levels could also be stable under field conditions as a non-invasive marker of disease progression (Heistermann and Higham, 2015).

Extensive clinical data was missing to investigate the impact of chronic inflammation from other coinfections (Trypanosomiasis, helminths, etc.) on the levels of neopterin as well as sCD40L, but these results suggest some degree of additional immune cell activation during an asymptomatic *Leishmania* infection in HIV patients. It remains to be seen whether such a high T-cell activation environment could be beneficial in the initial stages of infection and whether this remains true in chronic relapse cases. The value of neopterin as an alternative test of cure in VL/HIV was not investigated here. Because neopterin is only produced by humans and primates, literature in experimental models of VL is nonexistent. To our knowledge, only two older studies from the 90's and one recent study investigated the value of serum neopterin levels as markers of cure during treatment in CL and VL patients (Schriefer et al., 1995; Hamerlinck et al., 2000). Serum levels only appeared increased in VL patients before treatment, indicating a restricted association with a systemic infection. Only 1 out of 7 patients followed for 6–12 months after treatment died of leishmaniasis and showed a gradual increase in levels of neopterin. Vice versa, values in the other 6 patients decreased to normal values during treatment. These results confirmed the findings in 20 VL patients by Schriefer and coworkers (Schriefer et al., 1995). Likewise, Kip and coworkers recently confirmed the pharmacodynamic potential of neopterin to identify Sudanese and Kenyan VL patients at risk for VL relapse (Kip et al., 2018). Unfortunately, the longitudinal evaluation of neopterin in coinfected patients has not yet been reported.

Both molecules should be further explored as useful markers in a clinical algorithm for indirectly monitoring and predicting initial *Leishmania* infection progression in HIV patients, as recently proposed by Van Griensven and coworkers (van Griensven et al., 2014a). Antibody positivity has been suggested as an indicator of poor control (Th2 response). Nevertheless, all but one *Leishmania* antibody positive HIV patients remained asymptomatic for a median follow-up time of 1 year. To evaluate this hypothesis, longitudinal observational cohort studies with a large number of HIV patients in care living in endemic regions should comprehensively study asymptomatic infection with markers of Th1 immunity (Leishmanin Skin Test, T-cell functionality, etc.), Th2 immunity (antibody-based tests, etc.) as well as antigen detection (KAtex, Loop-mediated isothermal amplication (LAMP), RT-PCR, etc.), to discriminate past, latent or active *Leishmania* infection. Such studies would allow a simultaneous investigation of the utility of these cytokines and derive cut-off levels to suggest progression or resistance to VL. Advantages of cytokine measurements is that they allow for easy sample collection, analysis at low cost and require little technical competence, potentially using a partial or fully automated ELISA procedure.

## Conclusion

Our results match with the stated protective role of sCD40L in VL and indicate the importance of the CD40-CD40L pathway in early human immune responses against leishmaniasis, also in CD4+CD40L+ deprived HIV patients. Recombinant sCD40L could counteract the parasite's regulatory influence on host immunity and should be further explored with regard to resistance to *Leishmania* infection. On the other hand, neopterin levels could indicate general progression of disease, although non-specifically, and could be explored as a marker of a prognostic algorithm to predict VL progression in HIV patients.

## Ethics Statement

This study was carried out in accordance with the recommendations of the Declaration of Helsinki 2013, the Good Clinical Practice of the WHO, and those of the Ethiopian Food, Medicine and HealthCare Administration and Control Authority (FMHACA) with written informed consent from all subjects. The protocol was approved by the National Research Ethics Review Committee of Ethiopia, the University of Gondar Institutional Review Board (IRB), the Ethics Review Board of Médécins Sans Frontiers, the IRB of the Institute of Tropical Medicine, Antwerp and the Ethics Committee of Antwerp University Hospital.

## Author Contributions

WA conceived the study and drafted the manuscript. SA, EA, and FV contributed in sample measurements and data acquisition. SvH, YG, BM, ED, and EA helped in sample collection and daily coordination. Interpretation of the data was done by WA, LK, ED, and JvG. SA, SvH, YG, ED, FV, BM, EA, LK, and JvG commented on the draft. All authors read and approved the final manuscript.

### Conflict of Interest Statement

The authors declare that the research was conducted in the absence of any commercial or financial relationships that could be construed as a potential conflict of interest.
